# TRPV4 channels regulate tumor angiogenesis via modulation of Rho/Rho kinase pathway

**DOI:** 10.18632/oncotarget.8405

**Published:** 2016-03-26

**Authors:** Roslin J. Thoppil, Holly C. Cappelli, Ravi K. Adapala, Anantha K. Kanugula, Sailaja Paruchuri, Charles K. Thodeti

**Affiliations:** ^1^ Department of Integrative Medical Sciences, Northeast Ohio Medical University, OH 44272, Rootstown, USA; ^2^ School of Biomedical Sciences, Kent State University, OH 44240, Kent, USA; ^3^ Department of Chemistry, University of Akron, OH 44325, Akron, USA

**Keywords:** endothelial cell, mechanotransduction, Rho/Rho kinase, TRPV4, tumor angiogenesis

## Abstract

Targeting angiogenesis is considered a promising therapy for cancer. Besides curtailing soluble factor mediated tumor angiogenesis, understanding the unexplored regulation of angiogenesis by mechanical cues may lead to the identification of novel therapeutic targets. We have recently shown that expression and activity of mechanosensitive ion channel transient receptor potential vanilloid 4 (TRPV4) is suppressed in tumor endothelial cells and restoring TRPV4 expression or activation induces vascular normalization and improves cancer therapy. However, the molecular mechanism(s) by which TRPV4 modulates angiogenesis are still in their infancy. To explore how TRPV4 regulates angiogenesis, we have employed TRPV4 null endothelial cells (TRPV4KO EC) and TRPV4KO mice. We found that absence of TRPV4 (TRPV4KO EC) resulted in a significant increase in proliferation, migration, and abnormal tube formation *in vitro* when compared to WT EC. Concomitantly, sprouting angiogenesis *ex vivo* and vascular growth *in vivo* was enhanced in TRPV4KO mice. Mechanistically, we observed that loss of TRPV4 leads to a significant increase in basal Rho activity in TRPV4KO EC that corresponded to their aberrant mechanosensitivity on varying stiffness ECM gels. Importantly, pharmacological inhibition of the Rho/Rho kinase pathway by Y-27632 normalized abnormal mechanosensitivity and angiogenesis exhibited by TRPV4KO EC *in vitro*. Finally, Y-27632 treatment increased pericyte coverage and in conjunction with Cisplatin, significantly reduced tumor growth in TRPV4KO mice. Taken together, these data suggest that TRPV4 regulates angiogenesis endogenously via modulation of EC mechanosensitivity through the Rho/Rho kinase pathway and can serve as a potential therapeutic target for cancer therapy.

## INTRODUCTION

A key hallmark in the growth and progression of solid tumors is the sustained stimulation of angiogenesis [1]. Comprised of complex and tightly regulated processes, including basement membrane remodeling, endothelial cell (EC) proliferation, migration, and tube formation, angiogenesis plays a critical role in cancer. From the beginning of tumorigenesis, tumor cells cultivate an environment with an abundance of pro-angiogenic factors to allow for the formation of blood vessels that can infiltrate the solid tumor [2–3]. Tumor angiogenesis is an important control point for the progression of any solid tumor however, the resulting vasculature is highly abnormal, making the delivery of anti-cancer therapies difficult [4]. These challenges led to the development of anti-angiogenic therapies, which aim to target tumor angiogenesis as opposed to solely suppressing tumor growth. Many of these studies have focused on pro-angiogenic signaling pathways but have been met with limited success due to acquired resistance and/or impaired drug delivery, as well as their adverse effects on normal tissue [5–6]. Therefore, the identification of alternative therapies that target tumor angiogenesis are still of high value.

Emerging evidence has shown that the transient receptor potential (TRP) superfamily of ion channels is associated with a variety of cancers [7]. Aberrant functions of these channels can lead to sustained proliferative signaling, evading growth suppressors, resisting cell death, and increased production and secretion of mitogens, which are closely related with the defined hallmarks of cancer [8–11]. Further, many of these cell processes are highly sensitive to changes in [Ca^2+^]_i_ which makes TRP channels an attractive avenue to explore. TRPV4 channels play an important role in regulating EC physiology via mechanotransduction [12–19]. Our latest findings revealed that decreased functional expression of TRPV4 plays a role in the aberrant mechanical properties of TEC (tumor endothelial cells), which we were able to rescue with overexpression or pharmacological activation of TRPV4. We further demonstrated that TRPV4 activation normalized the tumor vasculature in WT mice injected with tumors, to promote efficient chemotherapeutic drug delivery and reduce overall tumor growth [12]. Taken together, these findings provide compelling evidence that TRPV4 influences angiogenesis however, the molecular mechanism by which TRPV4 regulates angiogenesis remains unknown.

In recent years, the Rho/Rho kinase pathway has become an attractive target in cancer medicine due to its involvement in cellular processes such as proliferation, cell shape, motility, as well as its contribution to tumor progression [20]. Several studies have shown that inhibition of the Rho/Rho kinase signaling pathway can decrease tumor cell proliferation and invasion *in vitro* as well as decrease tumor growth and metastasis *in vivo* [21–26]. Further, the Rho/Rho kinase pathway has been found to be involved in EC permeability, migration, and survival and has been found to play an integral part in vascular endothelial growth factor (VEGF)-mediated angiogenesis [27–28]. Although Rho/Rho kinase inhibitors have been used in a variety of cancer models to study tumor progression and/or metastasis [23, 29, 30], neither their effects on tumor angiogenesis and vascular normalization nor upstream regulators of this pathway have been identified. In the present study, we explored the molecular mechanism by which TRPV4 regulates angiogenesis by focusing on the Rho/Rho kinase pathway.

## RESULTS

### TRPV4 deletion induces increased EC proliferation, migration, and basal Rho activity

To determine the molecular mechanism by which TRPV4 may influence angiogenesis, we first isolated EC from the vascular sprouts that originated from WT and TRPV4KO aortic explants. EC were cultured in defined media and characterized by measuring the expression of endothelial cell markers, smooth muscle cell markers, and TRPV4, using immunofluorescence, Western blot, and qPCR analysis (Supplementary Figures S1 and S2). EC from both WT and TRPV4KO explants exhibited an endothelial phenotype in culture (Supplementary Figure S1A), as evidenced by the expression of EC marker CD31 (Supplementary Figure S1B) and absence of smooth muscle cell marker, alpha-SMA (Supplementary Figure S1C), confirming that these are bonafide EC. Western blot analysis revealed TRPV4 specific bands (two bands, one below and above 100 kDa) in WT EC, which were absent in TRPV4KO EC (Supplementary Figure S2A, S2B).

**Figure 1:**
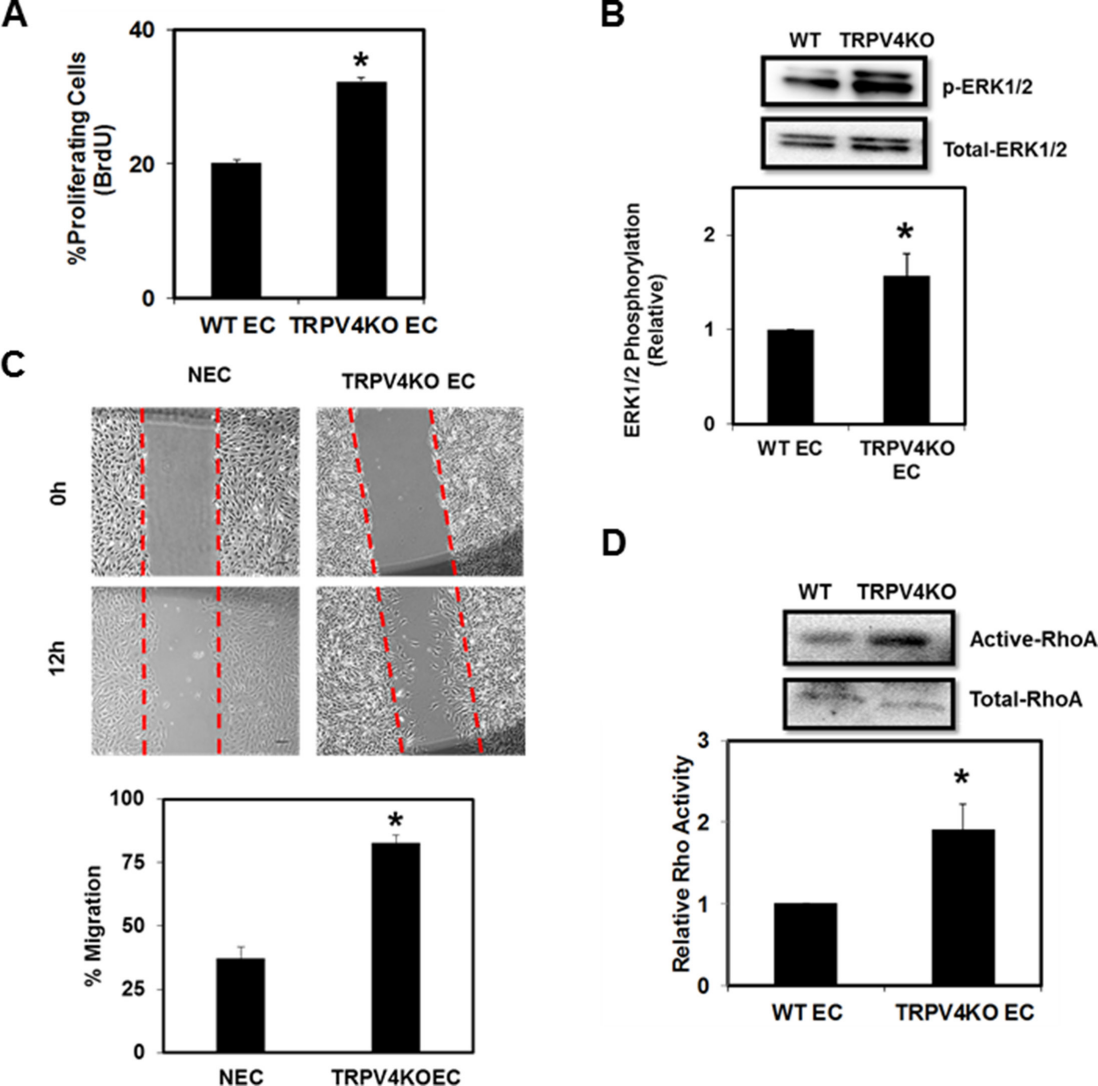
TRPV4 deletion induces abnormal EC proliferation, ERK1/2 phosphorylation, migration, and Rho activity (**A**) Quantitative analysis showing increased percentage of BrdU incorporation in TRPV4KO EC compared to WT EC. (**B**) Above: Western blot showing increased basal phosphorylation of ERK1/2 in TRPV4KO EC. Below: Densitometric analysis of ERK1/2 activity (normalized to total ERK1/2) showed a significant increase in TRPV4KO EC. (**C**) Above: Representative Brightfield images (4×) of scratch wounds taken at 0 and 12 hr. Scale bar = 100 μm. Below: Quantitative analysis showing increased migration of TRPV4KO EC compared to normal EC (NEC). (**D**) Above: Western blot showing increased basal levels of active RhoA in TRPV4KO EC compared to WT EC. Below: Densitometric analysis of RhoA activity (normalized to total RhoA) showed a significant increase in TRPV4KO EC.

EC proliferation and migration are important events in angiogenesis, which rely on the mechanosensing ability of EC to coordinate cytoskeletal reorganization and changes in cell adhesion [31–32]. Therefore, we hypothesized that lack of the mechanosensor TRPV4 would alter EC proliferation and migration. Indeed, we found that TRPV4KO EC showed increased proliferation (Figure 1A). We also found that this increased proliferation correlated with enhanced ERK1/2 phosphorylation in TRPV4KO EC (Figure 1B). Further, we found that TRPV4KO EC migrated significantly (*p* ≤ 0.001) greater than normal EC (NEC) (Figure 1C). These findings are reminiscent to the increased proliferation and migration we observed in tumor-derived EC (TEC) [12]. Furthermore, we have previously shown that high basal Rho activity, which controls the actin cytoskeleton, contributes to the abnormal mechanosensing ability of TEC, and that TRPV4 activation and/or overexpression can restore Rho activity to that comparable to NEC [12], suggesting that TRPV4 may regulate this pathway. Therefore, to explore whether the complete deletion of TRPV4 modulates EC migration via the Rho pathway, we performed Rho activity assays in WT EC and TRPV4KO EC. As hypothesized, we found that TRPV4KO EC exhibited significantly increased basal Rho activation (Figure 1D). Taken together, these results suggest that TRPV4 is required for the regulation of EC function by mediating optimal levels of Rho.

### Absence of TRPV4 induces abnormal angiogenesis *in vitro*, *ex vivo*, and *in vivo*

**Figure 2:**
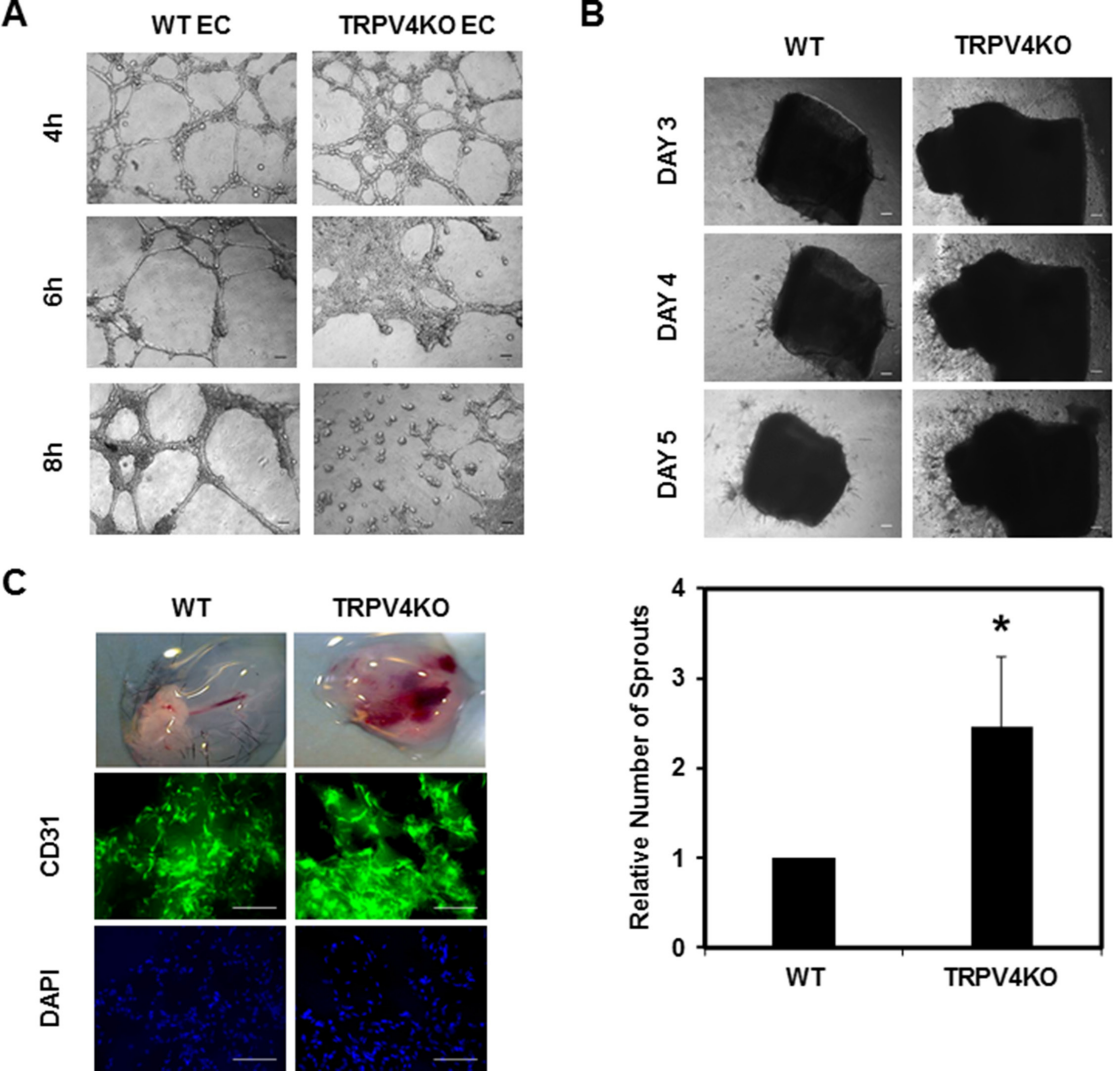
Absence of TRPV4 induces abnormal angiogenesis *in vitro, ex vivo*, and *in vivo* (**A**) Phase contrast images (4×) showing the angiogenic behavior of WT EC and TRPV4KO EC when plated on 2D Matrigel at high density (8 × 10^4^/well) at 4, 6, and 8 hours. (**B**) Above: Representative images (4×) showing sprouting angiogenesis from aortic explants isolated from WT and TRPV4KO mice. Below: Quantitative analysis showing significantly (*p* ≤ 0.05) increased vascular sprouting in aortic explants from TRPV4KO mice on Day 5. (**C**) Images of Matrigel plugs extracted from WT and TRPV4KO mice. Immunofluorescence images from Matrigel plugs sections (WT and TRPV4KO) showing vessel formation, as visualized by CD31 staining. Green *=* CD31; Blue = DAPI staining for nuclei. Scale bar = 100 μm for all images.

To investigate whether the inherent abnormalities associated with TRPV4KO EC (enhanced proliferation and migration) may also be translated in the way these cells form tubes, we performed 2D angiogenesis assays. We found that when plated on Matrigel, WT EC formed tubular structures within 4 h, which were stable until 8 h. In contrast, TRPV4KO EC formed tubes within 4 h, but then underwent multicellular retraction and tubular disruption to collapse after only 6 h (Figure 2A), suggesting abnormal angiogenesis by these cells.

To explore if TRPV4 expression also regulates angiogenesis, we examined vascular sprouting by performing *ex vivo* aortic ring assays. Using aortas isolated from WT and TRPV4KO mice, we incubated the aortic rings in Matrigel and monitored the growth of vascular sprouts for several days. We found that the aortic rings from TRPV4KO mice produced more sprouts and quantification of sprouts after 5 days of incubation showed significantly enhanced sprout formation from TRPV4KO compared to WT (Figure 2B). To further confirm these findings, we used *in vivo* Matrigel plug assays. Matrigel plugs supplemented with VEGF and FGF were subcutaneously injected into WT and TRPV4KO mice. Two weeks after the initial injection, Matrigel plugs were excised and the newly invaded endothelial cells were assessed for vessel formation by CD31 and DAPI staining (Figure 2C). Quantitative analysis of DAPI revealed an increase in the number of invaded cells, suggesting that EC may proliferate more in the TRPV4KO mice compared to WT (Supplementary Figure S3). Taken together, these data confirm that the absence of TRPV4 enhances angiogenesis *in vitro*, *ex vivo*, and *in vivo*.

### Pharmacological inhibition of the Rho/Rho kinase pathway restores mechanosensitivity and normalizes angiogenesis in TRPV4KO EC

**Figure 3:**
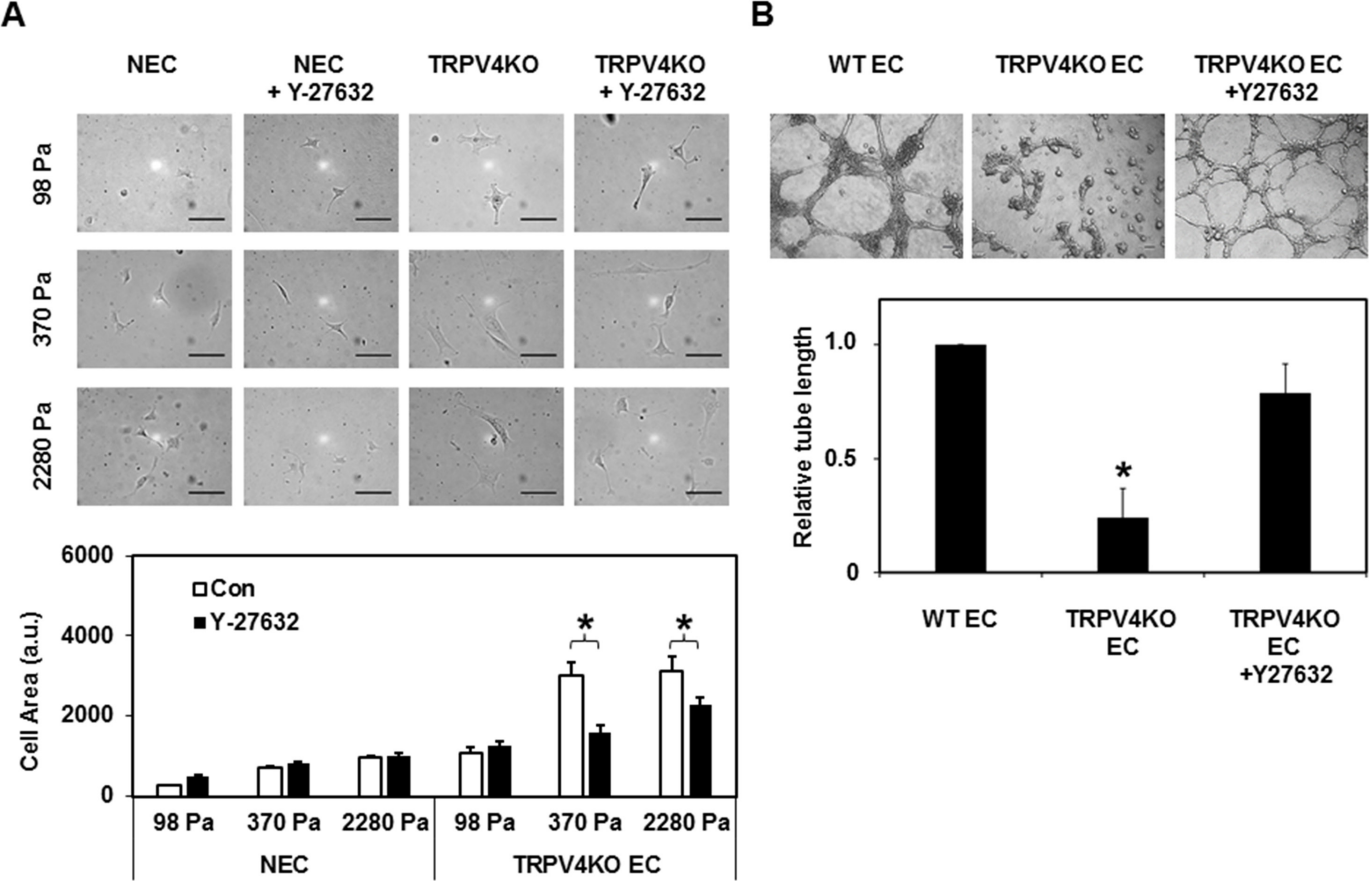
Rho kinase (ROCK) inhibition normalizes abnormal mechanosensitivity and angiogenesis exhibited by TRPV4KO EC (**A**) Above: Phase contrast micrographs (20X) showing the normalizing effects of Rho kinase inhibitor, Y-27632 (10 μM) on TRPV4KO EC spreading. Cells were equally plated on ECM gels of varying stiffness (98, 370, and 2288 Pa) and representative images were taken 6 h after plating. Scale bar = 100 μm. Below: Quantification of cell area revealed that while treatment with Y-27632 (10 μM) had no effect on NEC spreading, Y-27632 significantly attenuated abnormal TRPV4KO EC spreading at intermediate and high stiffness gels. (**B**) Above: Phase contrast micrographs (4×) showing the normalizing effects of Rho kinase inhibitor, Y-27632 (10 μM) on TRPV4KO EC angiogenic behavior, when plated on Matrigel at high density (8 × 10^4^ well). WT EC were used as a control. Representative images were taken 8 h post plating, showing the formation of stable tubes upon inhibition of the Rho kinase pathway. Scale bar = 100 μm. Below: Quantification of tube length revealed a significant increase (*p* ≤ 0.05) in tube formation in TRPV4KO EC+Y-27632 compared to untreated TRPV4KO EC controls. Further, the measured tube lengths of TRPV4KO EC treated with Y-27632 were comparable to WT EC.

The above findings suggest that in the absence of TRPV4, increased Rho/Rho kinase signaling may contribute to the aberrant mechanosensitivity of EC to ECM stiffness. To explore if the Rho/Rho kinase pathway influences TRPV4KO EC mechanosensitivity towards ECM stiffness, we next cultured NEC and TRPV4KO EC on transglutaminase linked gelatin gels of varying stiffness (98, 370, and 2280 Pa; representing low, intermediate, and high stiffness, respectively) [12, 33], which mimic the stiffness of tumor ECM [34–35]. Cells were seeded equally and allowed to spread for 6 h in the presence and absence of Y-27632 (10 μM). When we compared the degree of spreading of NEC, we found that the cell area increased from low to intermediate stiffness, and reached a plateau at the highest stiffness (Figure 3A), which was similar to our previous study [33]. On the other hand, while the TRPV4KO EC followed a similar trend, the spreading on intermediate and high stiffness gels was significantly more than at low stiffness (Figure 3A). Notably, treatment with Y-27632 reduced the cell spreading of TRPV4KO EC on both intermediate and high stiffness gels compared to untreated cells (Figure 3A), suggesting that pharmacological inhibition of Rho kinase with Y-27632 restored substrate mechanosensitivity in these cells. In contrast, Y-27632 had no significant effect on NEC spreading at low, intermediate, or high stiffness substrates. These results suggest that inhibition of the Rho/Rho kinase pathway restores mechanosensitivity in TRPV4KO EC.

Next, we asked if normalizing Rho or Rho kinase (ROCK; downstream effector of Rho) activity would normalize tube formation in TRPV4KO EC. To achieve this, we performed 2D angiogenesis experiments in the presence of Rho kinase inhibitor Y-27632 (10 μM) (Figure 3B). We found that in the absence of Y-27632, TRPV4KO EC formed tubes at earlier time points but collapsed after 6 h. However, these cells formed robust tubes in the presence of Y-27632, which were comparable to WT EC, stable after 8 h, and resulted in a significant increase in tube length (Figure 3B). Further, Y-27632 treatment had no significant effect on WT EC tube formation (data not shown). These results suggest that TRPV4 regulates angiogenesis via modulation of Rho signaling, and that the absence of TRPV4 results in abnormal angiogenesis.

### Rho kinase inhibitor Y-27632, in conjunction with Cisplatin, reduces tumor growth in TRPV4KO mice

**Figure 4:**
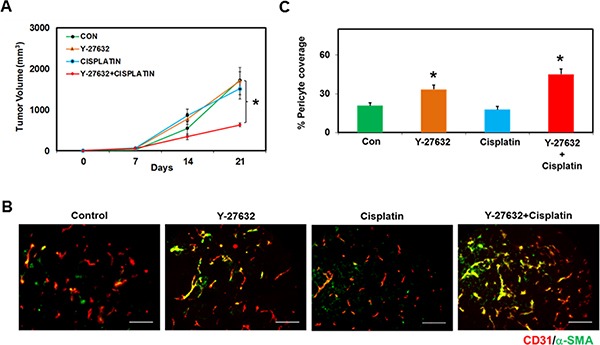
ROCK inhibitor, Y-27632, in conjunction with Cisplatin, reduces tumor growth *in vivo* in TRPV4KO mice (**A**) Time-dependent growth of tumors in mice injected with saline (CON), Y-27632, Cisplatin, or Y-27632 + Cisplatin. Syngeneic tumors (LLC) were injected in the back of TRPV4KO mice and tumor growth was monitored for 21 days. Rho kinase inhibitor, Y-27632, was injected (i.p.) (10 mg/kg) every day starting from Day 7 until Day 21. Cisplatin was injected (i.p.) (3 mg/kg) once per week starting 2–4 days after the injection of Y-27632. Note that tumor growth was significantly reduced in Y-27632+Cisplatin treated mice, but not Y-27632 or Cisplatin alone. (**B**) Frozen sections of tumors from Control, Y-27632, Cisplatin, and Y-27632+Cisplatin treated mice (10 μm thickness; from Day 21) were stained with CD31 (red) and α-SMA (green) to measure pericyte coverage (matured vessels). Scale bar = 100 μm. (**C**) Quantitative analysis of pericyte covered micro-vessels showing increased pericyte coverage in the tumor vessels treated with Y-27632 or Y-27632 + Cisplatin, but not in Cisplatin alone treated mice.

Our results clearly demonstrate that deletion of TRPV4 (TRPV4KO EC) results in increased Rho activation and angiogenesis *in vitro*, *ex vivo*, and *in vivo*. Further, our *in vitro* results showed that inhibition of the Rho pathway with Y-27632 normalized abnormal EC function and angiogenesis. Based on these findings, we hypothesized that Y-27632 treatment may reduce tumor growth in TRPV4KO mice. To accomplish this, we injected Lewis Lung Carcinoma (LLC) cells into TRPV4KO mice. Once the tumors became palpable (after ~7 days), we injected Y-27632 (i.p., 10 mg/kg) every day for 14 days. Saline injected mice served as controls. Additionally, to examine if Rho kinase inhibition normalizes tumor vasculature and improves cancer therapy, we treated mice with anti-cancer drug, Cisplatin (i.p., 3 mg/kg), once per week, alone and in combination with Y-27632. When used in combination with Y-27632, Cisplatin was administered 2–4 days after Y-27632 treatment. Overall, the TRPV4KO mice were divided into four groups: 1) Control 2) Y-27632 3) Cisplatin and 4) Y-27632 + Cisplatin. Tumor growth and angiogenesis was monitored as previously described [12]. We found that time dependent tumor growth in the control animals reached around 2000 mm^3^ at 21 days, consistent with our previous findings in TRPV4KO mice [12]. Interestingly, treatment with Y-27632 or Cisplatin alone did not have an effect on overall tumor size, but when used in combination, we found a significant reduction in tumor growth (Figure 4A). These results also suggest that Rho kinase inhibition may have normalized the tumor vasculature, which aided in the efficient and systemic delivery of Cisplatin. Consistent with this observation, we found increased pericyte coverage in tumor vessels that were treated with Y-27632 (Y-27632 and Y-27632 + Cisplatin), but not in the control or Cisplatin alone treated mice (Figure 4B and 4C). Together, our data provides compelling evidence that the Rho/Rho kinase pathway acts downstream of TRPV4 and that TRPV4 modulates angiogenesis by maintaining optimal levels of Rho/Rho kinase activity.

## DISCUSSION

Current and emerging anti-angiogenic therapies are still focused on growth factor signaling pathways, and while there has been therapeutic effects seen in mouse models and some human cancers, the clinical benefits are transitory at best, due to the inevitable development of resistance to the drug [36–37]. Therefore, the need to find alternative therapeutic approaches still remains. Calcium plays a multi-faceted role within the cell, and its involvement in regulating angiogenic processes makes it an important contributor, as well as potential molecular target, for tumor neovascularization and progression [38–40]. Recent reports suggest that transient receptor potential (TRP) channels, many of which are calcium permeable, play a crucial role in the biology of the tumor [41] and their expression in vascular endothelial cells may contribute in regulating tumor angiogenesis. In fact, it has been implicated that members from the TRPC and TRPV subfamilies are involved in endothelial function. TRPC1 and TRPC6 have been found to mediate pro-angiogenic effects by playing a role in VEGF-induced calcium influx [42–44]. More specifically, TRPC6 expression in HUVECs is critical for VEGF-induced proliferation and tube formation *in vitro* [45]. Moreover, TRPV1 has been found to play a pro-angiogenic role *in vivo*, where Yu *et al.* found that TRPV1 knockdown in zebrafish resulted in severe angiogenic defects [44]. While these channels have been identified to play a role in endothelial function and angiogenesis, little to no studies have implicated a specific role for these channels in the tumor vasculature.

TRPV4 is a ubiquitously expressed mechanosensor of shear stress and cyclic strain in the vascular endothelium [15, 16, 18, 19, 46]. Kohler and colleagues have demonstrated that TRPV4 is critical for sensing shear stress and endothelium-dependent vasodilation [14, 15, 47]. We have also shown that TRPV4 mediates cyclic strain-induced EC reorientation via integrin to integrin signaling [19]. Further, we found that EC derived from the tumor (TEC) fail to reorient in response to cyclic strain and exhibit abnormal angiogenesis *in vitro* [33]. Recently, we found low functional levels of TRPV4 in TEC, which may contribute to constitutively active Rho, abnormal mechanosensing, and tumor angiogenesis exhibited by these cells. Indeed, our latest studies demonstrated that directly targeting TRPV4 through pharmacological activation normalizes tumor angiogenesis and improves cancer therapy *in vivo* [12]. However, the precise mechanism by which TRPV4 may regulate angiogenesis remains to be elucidated.

In this study, we provided evidence that absence of mechanosensitive ion channel, TRPV4, acts as a negative regulator of angiogenesis. Recent studies have shown that TRPV4 deficiency leads to increased proliferation of different cell types, including renal duct cells and cholangiocytes [48, 49], and our previous study demonstrated that TRPV4-deficient TEC exhibit increased proliferation and migration [12, 50]. Therefore, we sought to explore how the complete absence of TRPV4 affected these processes. Here, our results show EC isolated from TRPV4KO mice clearly displayed increased proliferation and migration as well as abnormal tube formation. Further our *ex vivo* and *in vivo* results showed an increase in vascular sprouting from aortic rings as well as vascular growth in Matrigel plugs that were isolated from TRPV4KO mice, supporting the idea that TRPV4 negatively regulates EC function during angiogenesis

The Rho/Rho kinase pathway also plays an essential role in EC function and angiogenesis by regulating focal adhesion and stress fiber formation during cell migration [51–54]. Further, the tumor microenvironment contains vasculature surrounded by stiff tissue and ECM resulting in increased Rho/Rho kinase signaling in order to balance the external force with the internal cytoskeletal structure [55]. As a result, recent studies have begun investigate this pathway as a potential strategy to prevent tumor progression [28, 56–58] and inhibition of Rho kinase has started to show promise as a vascular normalizing agent. Therefore, to determine if the Rho/Rho kinase pathway is modulated by TRPV4-dependent mechanotransduction, we measured Rho activity and found that the absence of TRPV4 significantly increases basal Rho, suggesting that TRPV4 may regulates tumor angiogenesis by maintaining optimal levels of Rho.

Therefore, we next explored tube formation using 2D angiogenesis assays. When comparing WT EC and TRPV4KO EC, we found that TRPV4KO EC failed to stabilize and collapsed, which corresponds to high basal Rho activity. When we treated the cells with Rho/Rho kinase pathway inhibitor, Y-27632, we were able to normalize the abnormal angiogenesis displayed by TRPV4KO EC. Next, we asked if the Rho/Rho kinase pathway is the underlying molecular mechanism for enhanced tumor growth and tumor angiogenesis in TRPV4KO mice. We found that inhibition of the Rho/Rho kinase pathway *in vivo* in TRPV4KO mice treated with Y-27632 was able to normalize tumor vasculature, as evidenced by increased pericyte coverage of tumor vessels. This vascular normalization by Y-27632 in TRPV4KO tumors was further confirmed by reduced tumor growth when treated simultaneously with Y-27632 and Cisplatin.

**Figure 5:**
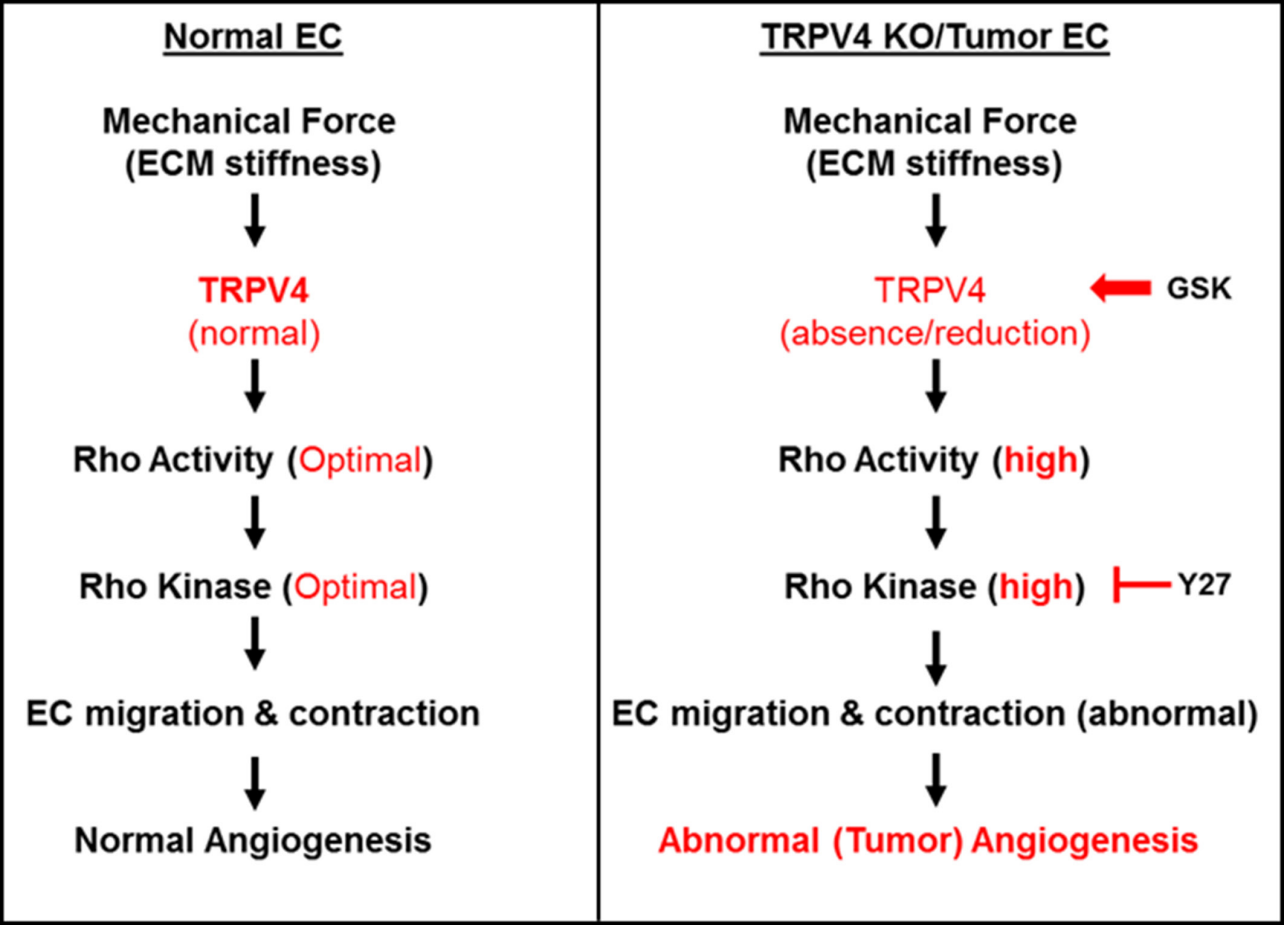
TRPV4-dependent mechanotransduction during angiogenesis A schematic representation of TRPV4-mediated mechanical signaling in normal and TRPV4KO/TRPV4-deficient (tumor) endothelial cells. In normal endothelial cells, TRPV4 senses mechanical force (ECM stiffness) and induces optimal Rho/Rho kinase activation necessary for endothelial migration and contraction which is required for partial cell rounding and angiogenesis. However, absence (TRPV4KO EC) or reduction (Tumor EC) in TRPV4 expression and function results in high basal Rho/Rho kinase activation, leading to abnormal (tumor) angiogenesis. This abnormal tumor vasculature can be normalized by restoring mechanosensitivity through pharmacological activation of TRPV4 in tumor endothelial cells (TRPV4-deficient) with GSK1016790A or inhibition of Rho kinase in TRPV4KO EC with Y-27632. Overall, these findings suggest that targeting TRPV4/Rho kinase-mediated mechanotransduction may be a novel therapy for tumor vascular normalization and improving anti-cancer drug delivery.

Recent work has started to highlight the role of mechanical forces in angiogenesis, as mechanical forces have been shown to modulate EC responses to growth factors. Importantly, changes in the balance between contractile (mechanical) forces generated by the cytoskeleton and the ECM regulate cell shape, proliferation, migration, and ultimately survival. In fact, cellular tension is sustained and regulated, to a large extent, by the Rho/Rho kinase pathway [31, 32, 59–61]. Although the role of the Rho/Rho kinase pathway has been studied extensively in response to growth factors such as VEGF [28, 62], upstream mechanotransduction pathways that regulate Rho/Rho kinase are not known. Our present study revealed that TRPV4, a mechanosensor in EC, is an endogenous upstream regulator of Rho/Rho kinase-dependent mechanotransduction and angiogenesis (Figure 5). Although anti-VEGF strategies have been suggested to be vascular normalizing agents, their success in clinics has been very limited. Importantly, recent PET imaging clearly demonstrated that anti-VEGF treatment rapidly (within 4 hours) attenuated perfusion to the tumors, which may act as a barrier to chemo and radiation therapy [63]. On the other hand, most of the work on the Rho/Rho kinase pathway is focused on targeting tumor cell proliferation and migration with little focus on tumor vasculature. Thus, our current findings propose that TRPV4 can be used as an alternate therapeutic target to inhibit/normalize tumor angiogenesis via modulation of Rho/Rho kinase pathway and improve cancer therapy.

## METHODS

### Cell culture

Cells from WT-C57BL/6 (WT) and TRPV4KO mice were isolated using the aortic explant method, as described previously [64–65]. Aortic rings were carefully removed from Matrigel after 7 days and EC were isolated, washed, and plated on 0.1% gelatin coated dishes. The isolated primary EC were cultured in a defined media, as previously described [12, 64], in a 37°C, 5% CO_2_ incubator, split at ~90–95% confluence, and used between passages 1–8. Cells were characterized for the presence of endothelial cell marker (CD31) and the absence of alpha-smooth muscle actin via Western blot, qPCR, and immunocytochemistry. Normal endothelial cells (NEC) were characterized and cultured as described previously [12, 33, 64, 66] and mouse Lewis Lung Carcinoma (LLC) cells were cultured in high glucose DMEM medium supplemented with 10% FBS and antibiotic/mycotic mix at 37°C.

### Aortic ring assay

Isolated aortic rings from WT and TRPV4KO mice, as previously described [64, 65], were carefully placed between 2 layers of Matrigel (BD Biosciences) and supplemented with defined endothelial cell medium. Aortic explants were monitored and imaged from Day 0 to Day 5 for vessel sprouting. The average number of sprouting vessels was calculated for Day 5 and data are expressed as fold increased from WT explants.

### Matrigel plug assay

WT and TRPV4KO mice were subcutaneously injected at the flank on both sides with growth factor-reduced Matrigel (Becton Dickinson), supplemented with basic fibroblast growth factor (bFGF; R&D Biosystems) (125 ng/plug), heparin sulfate (Sigma Aldrich) (15 μg/plug), and vascular endothelial growth factor (VEGF; R&D Systems) (1.2 ng/plug). Plugs were excised 2 weeks post implantation, fixed in 4% PFA, and sectioned for immunohistochemical analysis.

### BrdU proliferation assay

Cells were plated equally at low density on cover glasses for 24 hours, prior to treatment with 10 μM BrdU (Abcam) for 2 hours. Cells were then fixed in 4% PFA and stained as previously described [50]. Images were captured using an Olympus IX71-fluorcescence microscope and analyzed with ImageJ software (NIH). Quantitative analysis was performed by measuring BrdU positive cells in WT and TRPV4KO EC ((number of BrdU positive cells/total number of cells)*100).

### qPCR

RNA was isolated from WT and TRPV4KO EC using the RNeasy Mini Kit (Qiagen) and measured with the NanoDrop 2000 UV-Vis Spectrometer. cDNA was synthesized with qscript cDNA SuperMix (Quanta Biosciences) and the Fast SYBR green master mix (Applied Biosystems) was used for qPCR analysis on the Fast Real-Time PCR system (Applied Biosystems). The following real time primers were obtained from IDT technologies: CD31 and GAPDH. Gene expression was made relative to GAPDH and ΔΔCT values were expressed as fold change over WT.

### Western blot

Cells were lysed in Triton X-100, containing protease and phosphatase inhibitor cocktails (Boston Bioproducts). Lysates were loaded onto 8% or 10% acrylamide gels for separation via electrophoresis and transferred onto a PVDF membrane. The membrane was blocked in 5% milk in TBS with 0.1% Tween-20 (TBST), followed by overnight incubation with primary antibodies: anti-TRPV4 (1:300; Alomone Labs), tubulin (1:5000; Abcam), anti-phospho-ERK1/2 (1:1000; Cell Signaling), and total ERK1/2 (1:1000; Cell Signaling). After washing with TBST, membranes were incubated with the appropriate secondary antibodies, goat anti-rabbit (1:20,000) or goat anti-mouse (1:20,000), conjugated with horseradish peroxidase (Jackson Labs). Signals were detected with chemiluminescent substrates (Thermo Scientific) and developed with a FluorChem M Simple Imager (Protein Simple).

### Migration assay

Cells were plated on 6 well-plates and grown to 95% confluency. Cells were washed with PBS or serum free media to remove traces of growth factors. The scratch was made using a 200 μL tip and images were captured at 0 hour and 12 hours using an Olympus IX-51 brightfield microscope. Percent migration was quantified with Image J software (NIH) using the formula ((area of scratch a 0 hr – area of scratch at 12 hr)/area of scratch at 0 hr)*100)).

### 2D angiogenesis assay

Cells (8 × 10^4^ cells/well) were plated on growth factor-reduced Matrigel (BD Biosciences) in a 48 well-plate and kept at 37°C for up to 8 hours [12, 33]. For assays performed in the presence of Rho kinase (ROCK) inhibitor, cells were treated with Y-27632 (10 μM) prior to plating. Images were obtained with an Olympus IX-51 brightfield microscope and tube length was measured using Image J software (NIH).

### Rho activation assay

The Rhotekin-RBD affinity precipitation assay was used to analyze Rho activity, as previously described [12, 33, 67]. Briefly, cell lysates were incubated with GST-Rhotekin-RBD beads (Cytoskeleton Inc) for 1 hour at 4°C, followed by centrifugation and washing of the beads with wash buffer. Bound GTP-Rho was extracted with SDS sample buffer, probed for RhoA mAB (Santa Cruz) via Western blot, and GTP-Rho activity was analyzed from the resulting densitometric data. Active RhoA levels were normalized to total RhoA protein and values were expressed as fold change over WT.

### Tumor model

All animal experiments were performed according to an approved protocol by Northeast Ohio Medical University, IACUC. Mouse Lewis Lung Carcinoma (LLC) cells (2 × 10^6^) were injected into the flank on both sides of TRPV4KO mice [12]. Once tumors became palpable (after 7 days), six to eight mice/group were used for the following four treatment groups: (1) Control (2) Rho kinase inhibitor, Y-27632 (3) Cisplatin (4) Y-27632 + Cisplatin. Mice were given daily i.p. injections of Y-27632 (10 mg/kg) in groups 2 and 4. Anti-cancer drug, Cisplatin (3 mg/kg) was given once a week to group 3, and in group 4, Cisplatin was given once/week, 2–4 days post-treatment with Y-27632. Treatments were administered from Day 7 to Day 21, with the control group receiving saline as a vehicle. Calipers were used to measure tumor size at Day 7, Day 14, and Day 21 and tumor volumes were calculated from the formula (4/3*pi*length*((width/2)^2^)). Mice were sacrificed at Day 21 and tumor tissues were collected for immunohistochemical analysis.

### Immunohistochemistry

Matrigel plug or tumor tissue sections, cut at approximately 10 μM thickness, were fixed and permeabilized in ice cold acetone (20 min), washed with Tris buffered saline (TBS), and incubated overnight with anti-CD31 (1:50; Invitrogen) to visualize vessels. Tumor tissues were also stained for α-SMA (1:200; Sigma Aldrich) to visualize pericytes. Sections were washed, and incubated with the appropriate secondary antibody tagged with Alexa Fluor-488 or Alexa Fluor-594 (Invitrogen) and mounted with DAPI (Vector Labs). Images were captured with an Olympus IX-81 microscope and quantified with Image J software (NIH). Percent pericyte coverage was analyzed by dividing the number of CD31^+^/α-SMA^+^ cells by the total number of CD31^+^ cells multiplied by 100.

### Immunocytochemistry

WT and TRPV4KO EC were fixed in 4% paraformaldehyde (20–30 min), washed with PBS, and permeabilized with 0.25% Triton X-100 (15 min), followed by blocking with serum containing medium for 30–60 min at room temperature. Cells were then incubated with α-SMA (1:200; Sigma Aldrich) for 1 hour at room temperature or overnight at 4°C. Following washing, cells were incubated with Alexa Fluor-488 conjugated secondary antibody (1:500: Invitrogen) and mounted with DAPI (Vector Labs). Images were captured using an Olympus IX-71 fluorescence microscope. Lung fibroblasts were used as a positive control.

### Cell spreading on varying stiffness ECM substrates

Transglutaminase-crosslinked gelatin hydrogels of increasing stiffness were prepared using 3%, 5%, and 10% (w/v) final gelatin concentration, corresponding to 98, 370, and 2280 Pa, and incubated at 4°C overnight to stabilize crosslinking [12, 33]. Cells in regular culture medium were plated at low density (to minimize cell-cell interactions) and allowed to spread for 6 h. Images were captured using an Olympus IX-81 microscope and cell area was calculated using ImageJ software (NIH).

## SUPPLEMENTARY MATERIALS FIGURES


