# Supplementary material for: TRPV4 channels regulate tumor angiogenesis via modulation of Rho/Rho kinase pathway
Source: Oncotarget. 2016 Mar 26;7(18):25849–61. doi: 10.18632/oncotarget.8405 (PMC5041949; doi:10.18632/oncotarget.8405)
Supplement: Supplementary file 1 [file oncotarget-07-25849-s001.pdf]

TRPV4 channels regulate tumor angiogenesis via modulation of Rho/Rho kinase pathway

Supplementary Materials

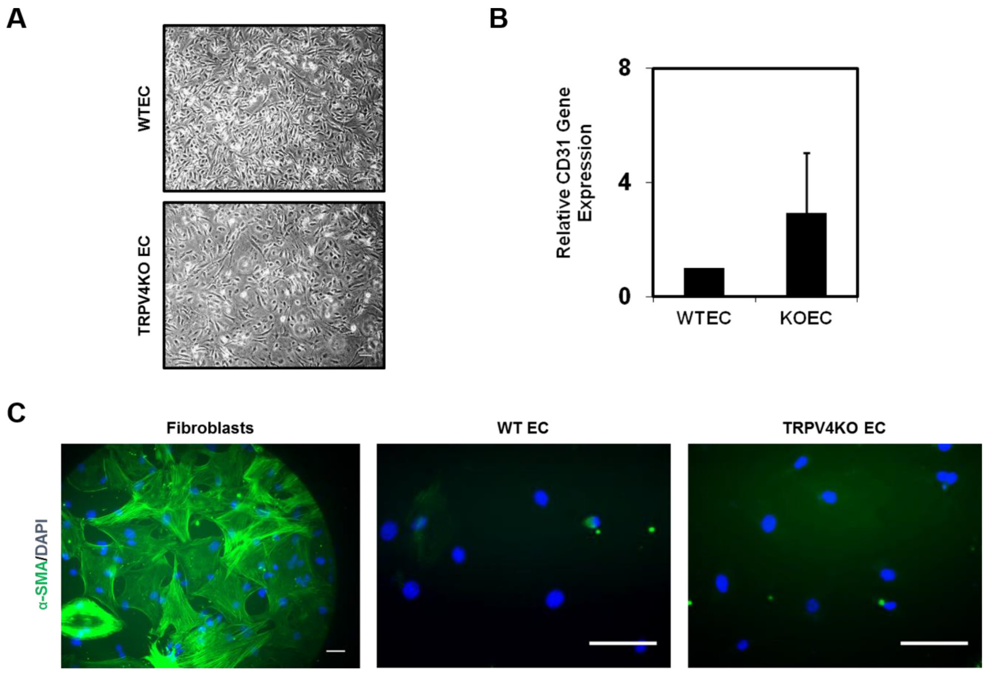

Supplementary Figure S1: Characterization of WT EC and TRPV4KO EC. (**A**) Photomicrographs of endothelial cells isolated from WT and TRPV4KO mice in culture. Scale bar = 100 µm. (**B**) qPCR analysis showing expression of endothelial marker, CD31 in EC. (**C**) Immunofluorescence images showing the absence of α-SMA in TRPV4KO and WT EC. Fibroblasts were used a positive control for α-SMA (green). Nuclei were stained with DAPI (blue). Scale bar = 100 µm.

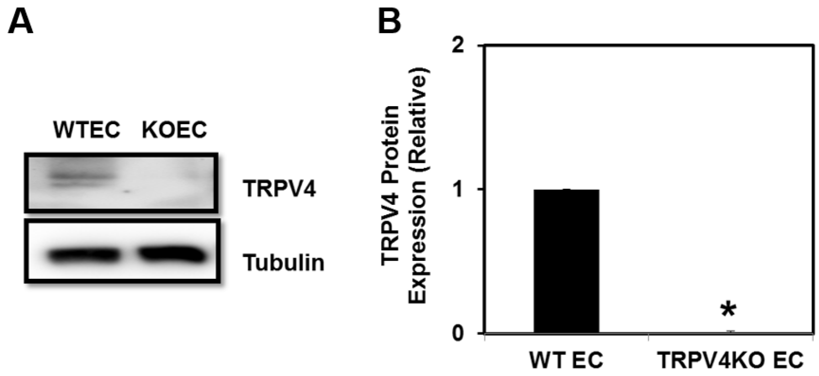

Supplementary Figure S2: TRPV4 expression in WTEC and TRPV4KO EC. (**A**) Representative Western blot showing TRPV4 protein levels in WT and TRPV4KO EC. (**B**) Densitometric analysis of western blots.

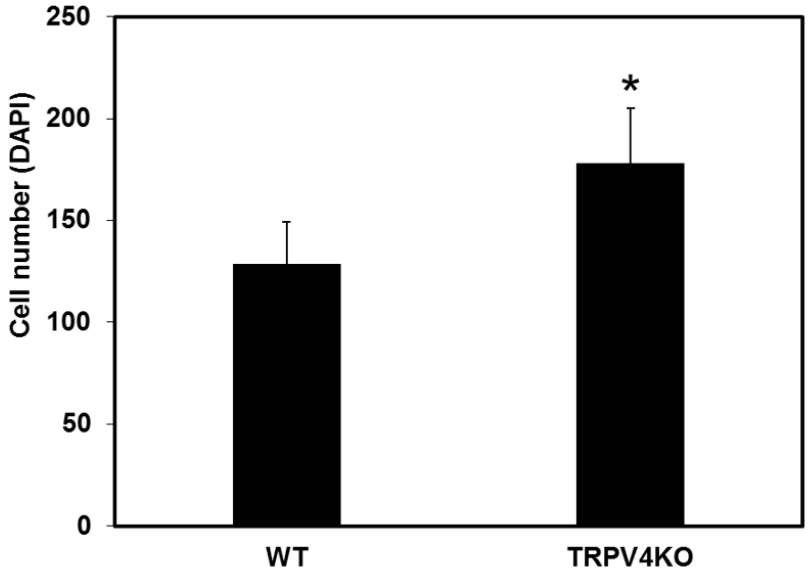

Supplementary Figure S3: Quantitative analysis of cell number (DAPI positive) from WT and TRPV4KO Matrigel plug sections (from Figure 2C; n = 6) ($p \leq 0.05$). All data shown is the mean ± SEM from at least three independent experiments.
